# Death of Dr. L. A. Dugas

**Published:** 1884-11

**Authors:** 


### DEATH OF DOCTOR L. A. DUGAS.

Dr. Louis A. Dugas died at his home in Augusta, on the 19th of October, after an illness of several days, at the advanced age of seventy-nine years.

Louis Alexander Dugas was born January 3, 1806, in Washington, Wilkes county, Ga., and was the son of Louis Rene Adrien Dugas de Vallon. The De Vallons were of French West Indian descent, immigrating from France some two generations ago to St. Domingo, where they became wealthy planters. His father, although born in St. Domingo, resided constantly in Paris, where his ample fortune enabled him to gratify his literary tastes; he was a gentleman of large and varied information, and had graduated in the law, besides acquiring great proficiency in the sciences. His mother, Mary Pauline Bellumeau de la Vincendiere, was a native of St. Domingo, where her parents had been wealthy planters for generations, but always educating their children in Paris, and it was there she met M. de Vallon and married him, in August, 1790. A gentleman of leisure and cultivated tastes—one of the old regime—had few inducements to remain in France in those troublous times, and accordingly M. de Vallon and his wife left for St. Domingo to settle on their plantation, where generations of their ancestors had preceded them. They had not been long there, however, before the revolution in the parent country extended itself to the colony, and resulted in the emancipation of the blacks, driving them with an infant daughter and but slender pecuniary means, to seek refuge in the United States. They landed in Charleston in 1791, and in deference to the republican simplicity of the land of their adoption, dropped the "de Vallon" from their name and were henceforth simply Dugas.

After about a year's residence in Georgetown, S. C., they removed to Newport, R. I., where they remained till 1801, when they removed to Frederickstown, Md. Three years in Maryland caused them to remove to Savannah, seeking a warmer climate, and in 1804 they settled in Washington, Wilkes county, where Dr. L. A. Dugas was born, with a twin brother, Louis Charles, who was afterwards a planter, and died in 1866 His father died in 1807, and in December, 1810, his mother, a most estimable and accomplished lady, removed to Augusta, established a female seminary, and was so successful as to educate her family and accumulate a competency. His mother educated her son until he was fifteen, with the exception of two or three quarters at the Academy of Richmond county. She had the proud satisfaction of seeing the prosperity of her children, and died in 1854, being then eighty-three years of age.

Dr. Dugas, in 1820, entered the office of Dr. Charles Lambert De Beauregard, a French emigré, to study medicine. Dr. De Beauregard dying in 1822, he entered the office of Dr. John Dent and studied for two years. He then attended lectures in Maryland and Philadelphia, and was graduated at the University of Maryland in March, 1827. The medical department of that University was then considered the best school in the country. He attended all the hospitals of the cities, and feeling the great responsibility of the practitioner he resolved to perfect himself in the European schools. After some plantation practice and study in Georgia, he sailed, in 1828, for Europe, where he remained upwards of three years, during which he made himself thoroughly acquainted with England, France, Switzerland, Germany and Italy, but making Paris his headquarters. There he devoted himself with persistent energies to his medical studies, devoting sixteen hours of each day to different branches of science, and so methodically arranging his time, alternating the severer with the more attractive branches of study, as to insure constant interest and avoid weariness. He attended lectures under such a galaxy of talent as Gay Lussac, Thenard, Pouillet, Arago, Cuvier, Blainville, Geoffry St. Hilaire, Beaumont, Majendie, Boyet, Roux, Velpeau, Dupuytren, Lesfranc, Cousin, Villemaine and Guizot.

During his stay in Paris he witnessed the great change from the deteriorating plan of treatment in diseases so strenuously advocated by Broussais, to the conservative system so successfully inaugurated by Andral, Louis, Rostan and others, and to which Dr. Dugas was then an enthusiastic and afterward a distinguished adherent. He also saw the inauguration of the Civiale system of lithotrity, and the development of that method of treament, of which he was the leading authority and practitioner in this country. He was present in Paris during the revolution of July, 1830, and was in the crowd at the Palais Royal when the troops first charged the people, and saw the first man killed. In June, 1831, he returned to America and commenced the practice of his profession in Augusta, Ga Soon after his arrival, the propriety of establishing a medical academy was mooted by Dr. Anthony, who asked Dr. Dugas to join him; this he agreed to do if the charter was altered to that of a medical college, and in 1832 the Medical College of Georgia was founded and organized, with Dr. Milton Anthony, Dr. Lewis D. Ford, Dr. Jos. A. Eve, Dr. Paul F. Eve, Dr. Jno. Dent and Dr. L. A. Dugas in the various chairs, the latter first taking that of Anatomy and Physiology. He some years afterwards yielded that of Anatomy to Dr. Geo. M. Newton, and taught Physiology added to Pathological Anatomy until 1855, when he took the Chair of the Principles and Practice of Surgery, which position he retained till two years ago, when he retired from active work, after a grand record of eminence in his profession and fifty years of service in the Medical College of Georgia, of which he was the distinguished and beloved Dean for twenty years.

In 1834 Dr. Dugas again visited Europe for the purpose of purchasing the library and general outfit for the museum of the Medical College. Each of the members of the faculty contributed $1,000, and for this sum, $6,000, he was enabled by his previous acquaintance with Parisian collections to obtain a fine museum, and was fortunate in securing, among other choice specimens, a "Cyclops," probably the finest example of that rare phenomenon, which could be readily sold for $1,000 to the British Museum to-day. In the library are some rare and valuable works purchased by him not often found in collections of this kind. While in Paris he was elected a member of the Geological Society of France. On his return to Augusta he paid especial attention to surgery, and in this branch of the profession his reputation is greater perhaps than any practitioner in this country. In 1851 he visited Europe a third time, and attended the first world's fair at the Crystal Palace in Hyde Park, London. The same year he returned and assumed editorial supervision of the *Southern Medical and Surgical Journal*, published in Augusta, which position he held seven years, making voluminous contributions to its pages. In addition to this literary work he contributed largely to other medical publications.

In the death of Dr. Dugas the medical profession of Georgia has lost its greatest ornament. For half a century as a practitioner, author and teacher, he stood in the sunlight of criticism, and has passed away without a single blot upon his bright escutcheon. To those who, like the writer, have for a large part of that period, been honored by his intimacy, the perfunctory words of eulogy sound cold and meaningless. The grandeur of his character cannot be summed up in a few phrases, or expressed by reference to a few incidents of his career. It can only be shown by the completeness of his entire life work; that is the office of his biographer.

In this brief notice the large number of his friends and former pupils amongst the readers of this journal will expect or pardon a reference to some of his professional peculiarities as a teacher. Perhaps the most noticeable of them was his devotion to truth. His extensive erudition, and his power of analysis, enabled him to detect the false and the speculative and fix upon the ascertained principles of every science to which he devoted himself. What seemed in his lectures extraordinary power of condensation was but the elimination of baseless figments of fancy from demonstrable facts. Untiring in his investigations, independent in his habits of thought and mental action, formed with judicial impartiality, his conclusions partook of the solidity of the solid rock, and their expression startled the listener by their exact accuracy and originalty.

As a surgeon the same habits of mind and patient labor gave to his opinions inestimable value. His diagnosis in all cases, especially in the doubtful and difficult ones, was proverbially unerring. In considering the propriety of an operation and in the execution of it, his care and provision were marvelous. No possible contingency was overlooked or unprovided for; no condition of success omitted, and the operative details in his hands absolutely perfect.

As a physician his scorn of shams and contempt for all imposture and false pretenses was characteristic. While illustrating in his own career the value of original investigation, in remedial measures and in operative procedures his rejection of startling novelties mere vagaries, the offspring of cupidity or vanity, was at once amusing and emphatic.

The great lesson of Dr. Dugas's life to the younger members of the profession is that it is possible for a man to be a physician and a gentleman. In England it is a proverb that no man can be engaged in trade and be a gentleman. Unfortunately the honorable profession of medicine has been degraded by alliance with the dirty usages of trade and the practice of the dubious devices of the shop or charlatan until the doubt is expressed whether the ideal honorable career of the physician be now practicable. To this doubt the noble and pure life of Dr. Dugas is a triumphant answer. No nobler gentleman ever trod the soil of Georgia; no stain of dishonor ever touched his garments; no falsehood in his own laudation or to another's detriment ever passed his lips; no arts of the harlequin ever disgraced his conduct. During a long life of unobtrusive usefulness his character was unsullied; every act of his life was beneficent; every throb of his heart was honorable. In every phase of his character he was a gentleman.

